# The Role of Digital Dermoscopy and Follow-Up in the Detection of Amelanotic/Hypomelanotic Melanoma in a Group of High-Risk Patients—Is It Useful?

**DOI:** 10.3390/life14091200

**Published:** 2024-09-22

**Authors:** Ružica Jurakić Tončić, Lara Vasari, Daška Štulhofer Buzina, Daniela Ledić Drvar, Mikela Petković, Romana Čeović

**Affiliations:** 1 Department of Dermatology and Venereology, University Hospital Center Zagreb, Kišpatićeva 12, 10000 Zagreb, Croatia; rjtoncic@gmail.com (R.J.T.); daniela.ledic.drvar@kbc-zagreb.hr (D.L.D.); dr.mikela@gmail.com (M.P.); romana.ceovic@gmail.com (R.Č.); 2 School of Medicine, University of Zagreb, Šalata 3, 10000 Zagreb, Croatia; 3 Naftalan Special Hospital for Medical Rehabilitation, Omladinska 23a, 10310 Ivanić-Grad, Croatia; lara.vasari@gmail.com

**Keywords:** digital dermoscopy, follow-up, amelanotic melanoma, hypomelanotic melanoma, high-risk patients

## Abstract

The prognosis, outcome, and overall survival of melanoma patients improve with early diagnosis which has been facilitated in the past few decades with the introduction of dermoscopy. Further advancements in dermoscopic research, coupled with skilled, educated dermatologists in dermoscopy, have contributed to timely diagnoses. However, detecting amelanotic and hypomelanotic melanoma remains a challenge even to the most skilled experts because these melanomas can mimic inflammatory diseases, numerous benign lesions, and non-melanoma skin cancers. The list of the possible differential diagnoses can be long. Melanoma prediction without the pigment relies only on vascular criteria, and all classic dermoscopic algorithms have failed to fulfill our expectations. In fact, the diagnosis of amelanotic and hypomelanotic melanomas is very challenging, which is why every tool in detecting these lesions is of significance. This review aims to explore the current knowledge and the literature on the possibility of detecting amelanotic/hypomelanotic melanomas using sequential monitoring with digital dermoscopy and total body skin photography.

## 1. Introduction

Monitoring patients with digital dermoscopy (DD) and total body skin photography (TBSP) may enable the detection of dynamic and subtle morphologic changes in cutaneous, nail, and even mucosal lesions with a focus on newly occurring lesions or recently changed lesions. Sequential monitoring with DD and TBSP has proved useful in minimizing the number of unnecessary excisions while at the same time pointing to suspicious lesions, thus giving rise to the possibility of early detection of melanoma [1]. The procedure is, however, time-consuming and requires trained and experienced dermatologists and expensive equipment. 

## 2. Indications for DD/TBSP Surveillance

This surveillance approach is not recommended for the general population and low-risk patients. A dermatologist skilled in early melanoma detection should select the group of high-risk patients. Data from a study conducted in 2018 showed that conventional handheld dermatoscopy was sufficient for melanoma detection in patients with solely a large number of (inconspicuous) nevi because, in this group of patients, more than 80% of melanomas were diagnosed for 10 years through a single dermatoscopic examination [2]. In their four-year monitoring study, Boroni et al. found that low-risk patients had no significant difference concerning the distribution of pT stages, mean Breslow tumor thickness, ulceration, and prevalence of associated melanocytic nevus in tumors diagnosed using periodical handheld dermoscopy compared to sequential DD [3]. In their study, the majority (60.67%) of melanomas diagnosed using DD were detected on first-time examination (baseline), indicating that in these low-risk patients, the capture and storage of digital dermoscopic images for further sequential DD would not have been necessary a priori [3]. Furthermore, after a median 24-month follow-up in a group of 145 low-risk patients without melanoma in personal history and patients with less than 50 melanocytic nevi, Schiffner et al. did not detect any melanoma by sequential DD, concluding that long-term monitoring with sequential DD seems to not help detect melanoma in this group of patients [4]. These data strongly indicate that selecting high-risk patients is crucial in detecting patients who can benefit from DD/total body skin photography (TBSP). 

## 3. High-Risk Melanoma Patients

High-risk groups for developing melanoma include individuals with genetic predisposition (CDKN2A mutation), those with skin that tends to burn (phototype I and II), people with inherited melanocortin-1 receptor (MC1R) variants, individuals with a high number of common nevi, those with large congenital nevi, multiple and/or atypical nevi, personal melanoma history, and individuals with intermittent high sun exposure [5]. Also at higher risk are patients with a family history of melanoma, because 5% to 10% of melanomas appear in melanoma-prone families who are likely to carry mutations in high penetrance susceptibility genes [5].

Total nevi count is the strongest independent risk factor, and the risk for melanoma increases almost linearly with a rising number of acquired melanocytic nevi [6]. Interestingly, Marghoob et al. found that total nevus count was inversely associated with melanoma thickness, meaning that patients with a higher nevus count had thinner melanomas and more melanomas in situ, independent of age and sex [7]. According to Rishpon et al., nevi with clinically atypical features also are among the strongest phenotypic risk factors [8]. Furthermore, even substantial changes in some dermoscopic features that are not specific to melanoma are considered a risk factor for melanoma [9]. Elder et al. presented a classification of nine ‘pathways’ based on clinical, histological, epidemiological, and genomic characteristics for developing cutaneous, mucosal, and uveal melanomas [10]. Considering clinical and histological morphological perspectives, as well as their genomic attributes, the authors describe nevi as potential melanoma simulants that need to be distinguished from melanomas by reliable diagnostic techniques [10]. Nevi are benign melanocytic neoplasms with mutations or fusions of the same single driver oncogenes that also occur in melanomas but lack the additional genomic changes [10]. All of the pathways shown in Table 1, Table 2 and Table 3 (adapted from Edler et al.) have an ‘intermediate’ category of lesions that have one or a few progression-related genomic changes (such as hemizygous loss of CDKN2A or a TERT promoter mutation) but insufficient to become melanoma [10]. Common driver oncogenes include mutations of BRAF or NRAS in cutaneous melanomas and others [10]. According to Elder et al., nodular melanomas, which are often amelanotic, likely occur in several of these pathways [10]. 

According to the International Dermoscopy Society, high-risk patients who benefit the most from DD and TBSP are patients with the indications listed in Table 4 [1]. The newest European consensus-based interdisciplinary guidelines for melanoma also state that all known high-risk groups might benefit from TBSP and that in high-risk individuals with multiple atypical nevi, sequential DD documentation facilitates both the detection of melanoma and a reduction in the number of unnecessary excisions [5].

Patients with primary melanoma in personal history could benefit from digital follow-up monitoring with DD/TBSP because of the higher risk for new primary melanoma [11]. According to the European guidelines, subsequent primary melanomas are diagnosed at a median of three years and are more often in situ and thinner than the initial tumors [5]. Guitera et al. report on a 9.0% risk annually for developing a new (secondary or tertiary) melanoma in the first 2 years of follow-up, which increased with time, particularly in those with multiple primary melanomas [11]. However, according to Garbe et al., the risk of subsequent melanoma decreases from 2% in the first year after diagnosis to a stable rate of 1% during the 15-year follow-up period [5]. A meta-analysis from Smith et al. identified several risk factors for the development of subsequent primary melanomas, as follows: CDKN2A mutation and high nevus count with high certainty of evidence, increasing age, presence of an atypical nevus, moderate nevus count with moderate certainty of evidence and male sex, family history of melanoma, light skin color, first lesions occurring on the head or neck, and inadequate sun protection with low certainty of evidence [12]. Porcar Saura et al. report on 69.2% and Haenssle et al. on 54.2% of melanomas associated with a pre-existing proliferation of nevus cells, respectively [13,14]. Additionally, Haenssle et al. found a significant association of nevi-associated melanomas with high nevi count (more than 100 common nevi), and 65.1% were found on the trunk [14]. Haenssle et al. conclude that patients with many acquired nevi on the trunk are at high risk for nevus-associated melanomas and may, therefore, particularly benefit from DD/TBSP surveillance [14]. Rishpon et al. also found a link between truncal nevi and melanoma risk, especially between anterior trunk (chest and abdomen) nevi and melanoma [8]. Smith et al. point out that better estimates of personal risk and improved risk stratification can assist with tailoring surveillance, guiding the use of TBSP and patient education, and also improving quality of life by reducing anxiety about developing melanoma [12]. To conclude, given all of the data, more detailed criteria are necessary to better determine high-risk groups of patients. 

## 4. Efficacy of DD/TBSP in Melanoma Detection

Sequential DD imaging involves capturing and assessing successive dermoscopic images separated by a time interval, of one or many melanocytic lesions to detect suspicious changes [15]. It is performed in two settings, short-term monitoring over three months for suspicious melanocytic lesions without dermoscopic evidence of melanoma, basically meaning selection of fast-growing lesions; and long-term monitoring for surveillance of multiple non-suspicious melanocytic lesions at intervals of six or twelve months [15]. According to Tschandl et al., a combined approach of short-term follow-up of lesions with an increased grade of atypia and long-term monitoring of larger numbers of inconspicuous lesions should achieve improved specificity and sensitivity for high-risk patients [16]. TBSP describes the use of clinical photography to provide a photographic record of a patient’s entire skin surface including 12–24 baseline photographs [15]. For example, FotoFinder ATBM takes 20 images of the body in eight assigned positions with one camera in about 10–15 min, and after TBSP, all suspicious lesions identified by a dermatologist are captured with the corresponding digital dermoscopy cameras (VISIOMED D200evo dermoscope, Medicam 1000) [17]. DD/TBSP surveillance also includes a handheld dermatoscopy of whole-body skin, which explains why these examinations are time-consuming. A cohort study by Salerni et al. showed that in high-risk patients, one-third of melanomas diagnosed during follow-up corresponded to lesions that were not under DD surveillance but were detected with TBSP [18]. An Australian study found that 38% of melanomas were diagnosed either exclusively or aided by TBSP, and 39% of melanomas were diagnosed with DD surveillance [19]. Over the last few years, studies investigated the value of surveillance with DD imaging, and the results suggest that approximately 10% of melanomas are detected only by changes in DD [9]. Other studies reported that 34–61% of the melanomas diagnosed in moderate-to-high-risk patients were detected by sequential DD surveillance [15]. Babino et al. report that approximately 60% of melanomas diagnosed by DD monitoring were morphologically featureless at the time of diagnosis and required a comparative assessment with previously captured images [9]. Furthermore, approximately 70% of 103 melanomas diagnosed by DD monitoring in their sample were in situ, and 30% were early invasive, with a mean Breslow thickness of 0.57 mm [9]. Also, in the study by Tshandl et al., all categories of melanomas, irrespective of whether they could be diagnosed at baseline or not, showed a median invasion thickness of much less than 1 mm with an overall median thickness of 0.4 mm, and many melanomas (48.3%) were still in situ [16]. In the study by Porcar Saura et al., the mean Breslow thickness was only 0.19 mm [13]. Guitera et al. found two-thirds (66.7%) of melanomas in their cohort due to observation of the significant changes in DD or TBSP which indicated biological activity [11]. Furthermore, in their cohort of 593 participants and 1513 excised lesions, the ratio of benign melanocytic lesions to melanoma excision was 2.4:1.0; and the ratio of melanoma in situ to invasive melanoma was 2.2:1.0 [11]. These data suggest that melanomas diagnosed by DD monitoring are thin melanomas, and therefore patients have better prognosis and survival outcomes. Moreover, in the study by Tshandl et al., the long interval from baseline examination until excision (median interval 15.4 months, range 3–133 months) indicates that most melanomas detected during follow-up showed a slow-growing progression pattern [16]. This is also the reason why follow-up of high-risk patients needs to be for long periods, if not lifelong. Tshandl et al. explain it by the fact that selected lesions for monitoring were mainly flat with a reticular pattern [16]. Also, they conclude that elevated or nodular lesions should not be included in follow-up examinations, but, when showing dermatoscopic atypia, they must be immediately excised [16]. According to Tschandl et al., other exclusion criteria for DD/TBSP monitoring are blue lesions, regressive lesions, lesions with a dermatoscopic clod pattern, and Spitzoid lesions, not including Reed nevi, as the latter can show fairly symmetric growth and stabilization [2]. 

## 5. Definition of Amelanotic/Hypomelanotic Melanoma

**Figure 1:**
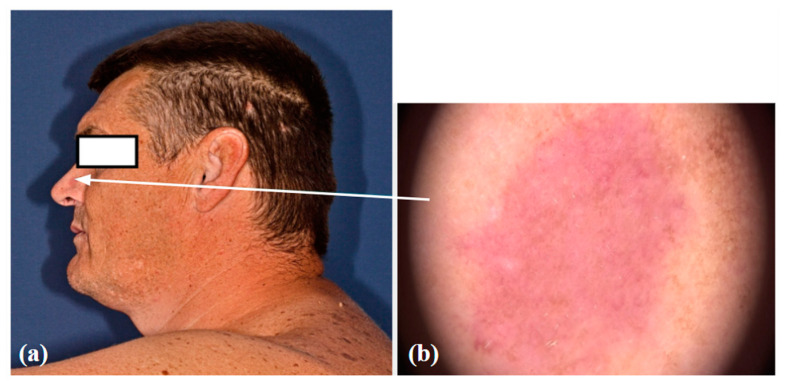
Hypomelanotic lentigo maligna: a clinical (**a**) and dermoscopic image (**b**).

**Figure 2:**
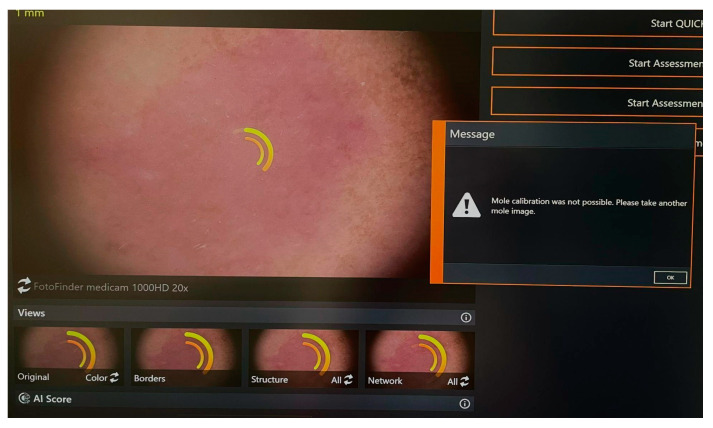
Lesion unable to be analyzed with Fotofinder.

**Figure 3:**
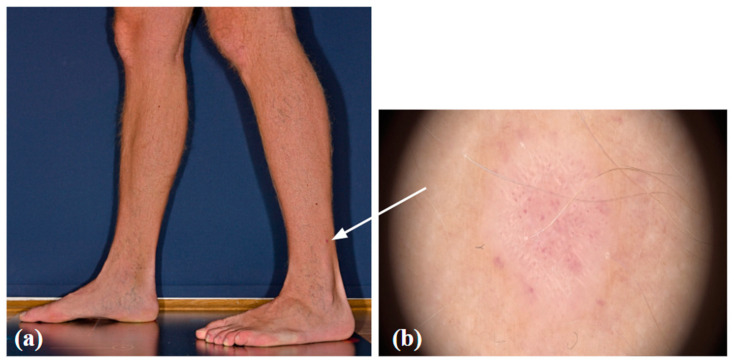
Amelanotic melanoma: a clinical (**a**) and dermoscopic image (**b**).

Amelanotic/hypomelanotic melanomas are a completely non-pigmented or only slightly pigmented subtype of melanoma, the early detection of which is still delayed resulting in poor prognosis [20]. Clinically amelanotic melanoma could be hypomelanotic upon dermoscopy. Definitions of AHM differ among studies [21]. Some authors define amelanotic melanomas as those that do not show any signs of melanin pigmentation under dermoscopy, whereas hypomelanotic melanomas may present as slightly pigmented without dark brown, deep blue, or black, or as partially pigmented lesions in which less than 25% of the total area shows melanin pigmentation [21]. Other authors define truly amelanotic melanoma as only completely amelanotic clinically and upon dermoscopy, with melanin in less than 5% of tumor cells upon histological examination [21]. Clinically amelanotic melanomas represent ill- or well-defined pink-to-red macules, papules, plaques, or nodules, often with a history of change in size, color, and shape [22]. Due to the wide spectrum of differential diagnoses, AHMs are known as great masqueraders and still pose diagnostic difficulties in everyday work, since these can look like numerous benign lesions and are easily overlooked. AHMs can occur on all body sites but are more common in chronically sun-exposed areas and in the head or neck areas [20,23]. Studies have also found amelanotic melanoma to be associated with older age, more commonly in individuals over 50 years of age [21,23]. According to Gong et al., patients with freckles, lack of nevi on the back, a sun-sensitive phenotype, or previous amelanotic melanoma are more likely to develop one [21]. Thomas et al. report an 8% and Wee et al. a 9.5% frequency of histopathologic amelanotic melanoma, which is within the range from 2% to 20% of melanomas previously reported as amelanotic [20,21,23]. It is well known that nodular melanomas are more likely to be amelanotic than other melanoma types, but Wee et al. also report on lentigo maligna and desmoplastic melanoma types independently associated with clinical AHM [20]. We present a case of hypomelanotic lentigo maligna from our clinical practice detected with DD/TBSP. (Figure 1) Still, the FotoFinder was not able to analyze the lesion. (Figure 2) And, the other case from our clinical practice also detected with Fotofinder is amelanotic melanoma. (Figure 3) Both of the presented lesions were detected upon baseline examination. 

Lampitielii et al. assert that any clinical subtypes of cutaneous melanoma may be amelanotic, but desmoplastic melanoma is the most frequent type, and the subungual site is the most common localization [24]. Also, mucosal melanomas have been described as amelanotic. A systematic review by Bansal et al. revealed 55 cases of oral amelanotic melanomas with poor prognosis and a 6.25% possibility of survival after 5 years [25]. In up to 40% of patients, mucosal melanomas are amelanotic, and up to 20% present clinically as multifocal [26]. It could be assumed that mucosal melanomas are often amelanotic because mucosal melanocytes are dermal, and their role is not to protect keratinocytes. A multicenter, retrospective observational study of 140 pigmented mucosal lesions reported the presence of blue, gray, or white color as the strongest clue in differentiating between malignant and benign mucosal lesions by dermoscopy [27]. Amelanotic melanomas have been positively associated with increased Breslow thickness and higher mitotic rate [23]. Thomas et al. report the median thickness of amelanotic melanomas to be much greater (1.60 mm) than that of pigmented melanomas (0.68 mm) [23]. These data may indicate that AHM grows faster than pigmented melanomas and therefore are more invasive with poorer prognosis compared to the other types of melanomas. Also, these data could be due to later presentation and later detection of these melanoma types. This is another strong argument, stating that any helping tool in diagnosing these melanomas as early as possible is important [23].

## 6. Diagnosing Amelanotic/Hypomelanotic Melanoma 

Using dermoscopy, it is possible to visualize subtle pigment if present in AHM. Atypical vessels defined as multiple vessels, different according to size and morphology (linear, dotted, or hairpin), are often the only relevant dermoscopic findings [22]. Vessel morphology in amelanotic melanoma varies among different types of lesions [21]. Dotted vessels are predominantly found in flat lesions, while nodular lesions usually present with linear vessels [21]. Thicker lesions, with Breslow thickness >1 mm, often show a higher frequency of hairpin, large, central, and peripheral vessels [21]. Milky red areas/globules, white lines, and in thicker tumors ulceration or erosion are other non-vascular dermoscopic features that can help diagnose these lesions [28]. A small case series by Stojkovic-Filipovic and Kittler confirmed polymorphous vascular patterns with no specific arrangement of vessels as dermoscopic characteristics of AHM [28]. Furthermore, Zalaudek et al. conclude that a combination of irregularly sized linear vessels and dotted vessels with a central pink-to-white veil are the dermoscopic hallmarks of AHM [22]. In a review of 49 cases, Dawood et al. report on short white lines as common and helpful predictive features in diagnosing AHM [29]. A meta-analysis by Lan et al. found a 61% sensitivity and 90% specificity of dermoscopy, and the authors conclude that both dermoscopy and reflectance confocal microscopy offer good diagnostic accuracy with high specificity and moderate sensitivity in diagnosing AHM [30]. However, a meta-analysis indicated that both dermoscopy and reflectance confocal microscopy might miss AHM more easily than pigmented melanomas [30]. 

## 7. Amelanotic/Hypomelanotic Melanoma and DD/TBSP

When it comes to the role of digital follow-up with DD and TBSP in detecting AHM, the literature is sparse, and according to our knowledge, a study in the field of using DD/TBSP for timely diagnosis of AHM has not been conducted. Digital follow-up consisting of DD and TBSP combined with artificial intelligence (AI) relies on machine learning which refers to the use of algorithms on structured data to identify patterns to guide classification or prediction, and when encountering new data, algorithms can predict an outcome based on prior training [31]. Deep learning is a subset of machine learning that uses neural networks. The most accessible application of deep learning to diagnose melanoma uses clinical photographs [31]. In diagnosing melanoma, deep learning could be using pre-trained networks, or it could be training a convolutional neural network (CNN) on images sourced from large datasets (ImageNet and the International Skin Imaging Collaboration Archive) or individually created datasets and then providing clinical photographs as inputs for the CNN to diagnose [31]. MacLellan et al. in their study evaluated three different models in the diagnosis of melanoma and compared them to the performance of a dermatologist using teledermoscopy (84.5% sensitivity, 82.6% specificity) and the face-to-face evaluation by dermatologists (96.6% sensitivity, 32.2% specificity) [32]. While several deep learning models had comparable or lower diagnostic performance compared to teledermoscopy or face-to-face evaluation by a dermatologist, the best performing one was FotoFinder Moleanalyzer Pro with 88.1% sensitivity and 78.8% specificity [32]. Also, Cerminara et al. in a cohort of 72 melanomas report that dermatologists had a higher sensitivity of 96.6% but a lower specificity of 32.2% than Moleanalyzer Pro [17]. Real-world data on the potential of collaboration between dermatologists and AI assistance in melanoma detection in daily clinical practice are still lacking [17]. No studies are focusing only on using DD/TBSP in detecting AHMs because AHMs are rare and the world database of clinical and dermoscopic images is scarce. Moloney et al. in 311 patients with a median follow-up of 3.5 years found five melanomas post-baseline thicker than 1 mm, three of which were histologically desmoplastic and two of those desmoplastic melanomas were amelanotic on dermoscopy; the other two had both nodular and superficial spreading components and were clinically amelanotic although a dermoscopic image was not available to confirm it [19]. Of 171 new primary melanomas detected during follow-up surveillance, Guitera et al. found only 7 melanomas thicker than 1 mm, of which 2 were self-detected, and of the 5 detected by a clinician, 2 were detected with the aid of TBSP [11]. Datasets in the future should include labels with melanoma subtypes to aid in model development and possibly become helpful in diagnosing AHM [31]. In the future, DD/TBSP could be useful in detecting AHM, but currently, lacking the data, there is a high risk of false positives and false negatives. While DD and TBSP have significantly advanced melanoma detection, addressing false positives and negatives remains crucial. The ongoing improvements in technology, combined with better training and standardized protocols, are essential for maximizing the benefits of these tools and minimizing potential risks, ultimately improving patient outcomes.

Lastly, a study by Birkenfeld et al. demonstrated that wide-field photography combined with computer-aided classification systems can distinguish suspicious from non-suspicious pigmented lesions, and this approach could be useful in supporting skin screenings at a population level [33]. Furthermore, results by Soenksen et al. suggest that deep learning systems adapted for wide-field analysis are a feasible approach to provide full-body dermatological triaging of suspected pigmented lesions for primary care settings [34]. However, in their studies, only pigmented skin lesions were analyzed. Using this method in detecting AHM could also result in many false positive or false negative results. Recently, there have been efforts to introduce better and more comprehensive systems for total body photography in order to not miss lesions which are usually not shown in classical systems that are available on the market today. The authors describe in detail how these systems work, but still, there is a need for more clinical experience in everyday work [35,36,37]. 

## 8. Conclusions

Because of the lack of melanin pigment, AHMs pose a diagnostic challenge, and the prognosis is worse than for other melanoma subtypes. Dermoscopy and reflectance confocal microscopy are accurate additional tools that help in the timely diagnosis of these lesions. Currently, data about using DD/TBSP in the detection of AHM are missing. In the future, meta-analysis techniques could be used to search and combine data from multiple studies, which can increase the overall sample size and provide robust evidence of DD/TBSP usefulness in detecting AHM. We need more data on this under-reported type of melanoma. Hopefully, this paper might encourage new studies and new papers regarding this under-reported issue. 

The strongest limitation of this review is the current lack of data, and therefore it is not possible to make strong conclusions and recommendations. Current evidence does not support the claim that digital dermoscopy significantly improves the early detection of amelanotic/hypomelanotic melanomas in high-risk patients. 

In this paper, we did not address the possibility of false positive/false negative outcomes in DD since this review has been conducted with the main aim of seeing the potential of DD in improving our diagnostic skills in diagnosing AHM. 

When it comes to the criteria of the high-risk patients, propositions of the IDS are already listed in the review. Still, in our personal experience, AHM has been more commonly seen in fair-skinned individuals (skin type I) who are included in the group of high-risk patients. These patients are generally demanding of the clinician since in fair skin types, melanoma usually presents with less dermoscopic criteria.

Regarding the high expense and time-consuming nature of the DD/TBSP, we recommend this type of healthcare only for high-risk patients in specialized skin cancer centers. We did not consider any alternative diagnostic methods in terms of accuracy, cost, and practicality to DD and TBSP since the main focus was on the role of DD and TBSP. We encourage clinicians to share new opinions, experiences, and publications regarding the diagnosis of AHM. 

Furthermore, we did not consider the ethical considerations of implementing widespread use of DD/TBSP, particularly in terms of patient anxiety, overdiagnosis, and unnecessary biopsies and excisions, since we believe that this discussion is beyond the aim of this review. Nevertheless, a skilled clinician with experience in this kind of monitoring can have less unnecessary excisions and give the patient the possibility to monitor the lesions more closely. 

With more additional information and more images of these lesions for datasets, deep learning systems may become better at detecting AHM. The data so far indicate that deep learning systems are not refined to the point of making highly reliable diagnoses of melanoma, especially of any melanoma subtypes, including AHM, without a dermatologist’s input. However, surveillance of high-risk patients with DD/TBSP and deep learning systems helps diagnose melanomas at an early stage, which then improves prognosis and survival rates, enhances overall quality of life, and reduces long-term healthcare costs associated with advanced disease management. But, in the detection of AHM, future studies and meta-analyses are needed to determine the potential and effectiveness of DD/TBSP surveillance which would justify the cost-effectiveness of this method. 

## Figures and Tables

**Table 1:** Pathway I, adopted from the Edler et al. [10]. Low UV irradiation exposure/CSD.

Pathway	I			
Endpoint of pathway	Low-CSD melanoma/SSM			
Benign neoplasms	Nevus			
Intermediate lesion (LGD and melanocytomas)	LGD	BIN	DPN	
Intermediate lesion (HGD and melanocytomas)	HGD/MIS	BAP1-inactivated melanocytoma/ MELTUMP	Deep penetrating melanocytoma/ MELTUMP	PEM/MELTUMP
Malignant neoplasms	Low-CSD melanoma/SSM	Melanoma in BIN	Melanoma in DPN	Melanoma in PEM
Common mutations	BRAF p.V600E or NRAS TERT, CDKN2A, TP53, PTEN	BRAF or NRAS + BAP1	BRAF, MAP2K1 or NRAS + CTNNB1 or APC	BRAF + PRKAR1A or PRKCA

Abbreviations: BIN, BAP1-inactivated nevus; CSD, cumulative solar damage; DPN, deep penetrating nevus; LGD, low-grade dysplasia; HGD, high-grade dysplasia; MELTUMP, melanocytic tumor of uncertain malignant potential; MIS, melanoma in situ; PEM, pigmented epithelioid melanocytoma; SSM, superficial spreading melanoma.

**Table 2:** Pathways II and III, adopted from Elder et al. [10]. High UV radiation exposure/CSD.

Pathways	II	III
Endpoint of pathway	High-CSD melanoma/LMM	Desmpolastic melanoma
Benign neoplasms	? IMP	? IMP
Intermediate lesions (LGD and melanocytomas)	? IAMP/dsyplasia	? IAMP/dysplasia
Intraepidermal malignancies (HGD and melanocytomas)	Lentigo maligna (MIS)	MIS
Malignant neoplasms	LMM (VGP)	Desmoplastic melanoma
Common mutations	NRAS; BRAF (non-p.V600E); KIT; or NF1 TERT; CDKN2A; TP53; PTEN; RAC1	NF1; ERBB2; MAP2K1; MAP3K1; BRAF; EGFR; MET; TERT; NFKBIE; NRAS; PIK3CA; PTPN11

Abbreviations: CSD, cumulative solar damage; IAMP, intraepidermal atypical melanocytic proliferation; IMP, intraepidermal melanocytic proliferation without atypia; LGD, low-grade dysplasia; LMM, lentigo maligna melanoma; MIS, melanoma in situ; HGD, high-grade dysplasia; VGP, vertical growth phase (tumorigenic and/or mitogenic melanoma).

**Table 3:** Pathways IV–VIII, adopted from Elder et al. [10]. Low-to-no (or variable/incidental) UV radiation exposure/CSD.

Pathways	IV	V	VI	VII	VIII
Endpoint of pathway	Spitz melanoma	Acral melanoma	Mucosal melanoma	Melanoma in CN	Melanoma in BN
Benign neoplasms	Spitz nevus	? Acral nevus	? Melanosis	CN	BN
Intermediate lesions (LGD and melanocytomas)	Atypical Spitz tumor	IAMPUS/ dysplasia	Atypical melanosis/ dysplasia/ IAMPUS	Nodule in CN (melanocytoma)	(Atypical) cellular BN (melanocytoma)
Intermediate lesions (HGD and melanocytomas)	STUMP/ MELTUMP	Acral MIS	Mucosal MIS	MIS in CN	Atypical CBN
Malignant neoplasms	Spitz melanoma	Acral melanoma (VGP)	Mucosal lentiginous melanoma (VGP)	Melanoma in CN	Melanoma in blue nevus
Common mutations	HRAS; ALK; ROS1; RET; NTRK1; NTRK3; BRAF; or MET CDKN2A	KIT; NRAS; BRAF; HRAS; KRAS; NTRK3; ALK; or NF1 CDKN2A; TERT; CCND1; GAB2	KIT; NRAS; KRAS; or BRAF NF1; CDN2A; SF3B1; CCND1; CDK4; MDM2	NRAS; BRAF p.V600E (small lesions); or BRAF	GNAQ; GNA11; or CYSLTR2 BAP1; EIF1AX; SF3B1

Abbreviations: BN, blue nevus; CBN, cellular blue nevus; CN, congenital nevus; CSD, cumulative solar damage; IAMPUS, intraepidermal typical melanocytic proliferation of uncertain significance; MELTUMP, melanocytic tumor of uncertain malignant potential; MIS, melanoma in situ; STUMP, Spitzoid tumor of uncertain malignant potential; UV, ultraviolet; VGP, vertical growth phase (tumorigenic and/or mitogenic melanoma).

**Table 4:** List of indications for digital monitoring in patients with multiple nevi *.

Indications for DD/TBSP in Patients with Multiple Nevi
>60 melanocytic nevi
CDKN2A mutation or other rarer high-risk melanoma genetic variants
>40 melanocytic nevi and a personal history of melanoma
>40 melanocytic nevi and red hair and/or a MC1R mutation
>40 melanocytic nevi and a history of organ transplantation

* Adapted from Russo et al. [1].

## Data Availability

No new data were created or analyzed in this study. Data sharing is not applicable to this article.
